# Epileptic-Net: An Improved Epileptic Seizure Detection System Using Dense Convolutional Block with Attention Network from EEG

**DOI:** 10.3390/s22030728

**Published:** 2022-01-18

**Authors:** Md Shafiqul Islam, Keshav Thapa, Sung-Hyun Yang

**Affiliations:** Department of Electronics Engineering, Kwangwoon University, Seoul 139-701, Korea; msislam@kw.ac.kr (M.S.I.); kshavthapa@kw.ac.kr (K.T.)

**Keywords:** epileptic seizure (ES), electroencephalogram (EEG), convolutional neural network, feature attention module

## Abstract

Epilepsy is a complex neurological condition that affects a large number of people worldwide. Electroencephalography (EEG) measures the electrical activity of the brain and is widely used in epilepsy diagnosis, but it usually requires manual inspection, which can be hours long, by a neurologist. Several automatic systems have been proposed to detect epilepsy but still have some unsolved issues. In this study, we proposed a dynamic method using a deep learning model (Epileptic-Net) to detect an epileptic seizure. The proposed method is largely heterogeneous and comprised of the dense convolutional blocks (DCB), feature attention modules (FAM), residual blocks (RB), and hypercolumn technique (HT). Firstly, DCB is used to get the discriminative features from the EEG samples. Then, FAM extracts the essential features from the samples. After that, RB learns more vital parts as it entirely uses information in the convolutional layer. Finally, HT retains the efficient local features extracted from the layers situated at the different levels of the model. Its performance has been evaluated on the University of Bonn EEG dataset, divided into five distinct classes. The proposed Epileptic-Net achieves the average accuracy of 99.95% in the two-class classification, 99.98% in the three-class classification, 99.96% in the four-class classification, and 99.96% in classifying the complicated five-class problem. Thus the proposed approach shows more competitive results than the existing model to detect epileptic seizures. We also hope that this method can support experts in achieving objective and reliable results, lowering the misdiagnosis rate, and assisting in decision-making.

## 1. Introduction

The brain is the central organ of the nervous system and is responsible for controlling most body functions. Although the human brain is vital, different conditions like Alzheimer’s, dementia, brain cancer, epilepsy, other seizure disorders, Parkinson’s, stroke, and transient ischemic attack (TIA) have increased significantly. Epilepsy is the second most common chronic neural disease, which can affect people of all ages [1,2,3]. Human brain activity becomes aberrant due to epilepsy, resulting in seizures or periods of odd behavior, sensations, and sometimes lack of awareness. A seizure is a temporary disturbance in the electrical activity of brain that most usually begins with uncontrollable contractions and unresponsiveness in the muscles and may affect one or more body areas. The World Health Organization (WHO) reports that around 65 million individuals worldwide are suffering from epileptic seizures [2].

When a sudden abnormal and self-sustained electrical discharge happens in cerebral networks and sustains for less than a few minutes it is called an epileptic seizure (ES). It is quite hard to predict the ES attacks because the severity and attack duration is not easily presumed. So, there is a need to prevent the injuries and ensure the safety for patients. An early prediction of epilepsy attacks is advantageous to avoid the injuries and life threats.

There are several methods used to diagnose epilepsy, such as magnetic resonance imaging (MRI), electroencephalogram (EEG), and magneto-encephalography (MEG). Amongst the most practised methods, EEG is used [4] to record the brain’s electrical activity by placing electrodes in various positions on the scalp. EEG signal is non-Gaussian and non-stationary, can diagnose brain disorders. Recently expert neurologists examined a long-term recorded EEG signal to predict the threat of epilepsy. In order to get an accurate prediction of epilepsy, the patient had to undergo EEG recording over several days, weeks, or even months, which is uncomfortable for both the patient and expert side. Additionally, undue dependence on the experts of individuals and the subjective decision of neurologists can bring about various demonstrative outcomes for similar EEG segments. Therefore, a reliable and automatic procedure is needed to correct and automatic detection of epileptic seizures to resolve the above limitations.

The seizure detection system utilizes feature extraction and classification modules for automatic detection [5,6]. However, the existing automated seizure detection system uses conventional methods (e.g., hand-crafted features extracted from time-domain [7,8], frequency-domain [9,10,11], wavelet-domain [12,13], and sometimes from multiple domains [6,14]) and machine learning (ML) techniques. Nowadays, deep learning (DL) methods have emerged as an important research trend in biological signal-based ES detection. Deep neural networks (DNNs) are composed of different types of processing layers. It provides significant performance over the traditional handcraft based feature extraction method [15,16,17,18].

The performance of the model can be enhanced by introducing an attention mechanism to the spatial dimension which does not significantly increase the number of learning parameters. However, the attention mechanism with DL has not been adopted in ES detection based on the literature review. So, we have been motivated to introduce a feature attention method in ES detection, which can increase features representation power as well as comprehensibility. The proposed feature attention-based method blends dense convolutional blocks (DCB), feature attention modules (FAM), and residual blocks (RB) on a convolution neural network (CNN) model to detect ES. The proposed model is evaluated on the University of Bonn EEG dataset, and it outperforms the state-of-the-art methods for different problems relating to ES detection.

The main objectives of this study are: (i) To segment the raw EEG data by splitting it into many subsamples, which increases the number of samples using data augmentation and may help to train a deep model; (ii) to develop a deep 1-D CNN attention model to improve the classification accuracy than existing methods; (iii) to evaluate the performance of the data augmentation and the deep models for detecting different cases of epilepsy from dataset; and (iv) the performance comparison of the proposed method with well-established DL architectures adapted for ES Detection.

## 2. Related Work

Automatic seizure detection has been studied for decades, and several promising outcomes have been obtained. The journey of ES detection began in the early 1980s [19]. At an early stage, to detect epileptic seizures from EEG signal linear prediction [20,21], spectral estimation [22], and auto regression model [23] were used. However, due to the non-stationary and nonlinear character of the EEG signal, those methods were unable to provide a sufficient analysis of the signals. Therefore, time-frequency approaches decompose an EEG signal on both the time and frequency axes simultaneously, which gives information on how much energy is distributed throughout the different frequencies present in the signals. As a result, numerous time-frequency approaches, short-time Fourier transforms (STFT), wavelet transforms (WT), stockwell transforms (ST), empirical mode decomposition (EMD), variational mode decomposition (VMD), and many more have been used for analyzing non-stationary EEG signals.

Sharma [24] decomposed the input signals using EMD and collected several vital features from every intrinsic mode function (IMFs). After that, the least squares support vector machine (LS-SVM) was utilized to classify and achieved 95% accuracy for the two classes. Next, Zhang [25] decomposed the input EEG signals into several product functions (PFs) using local mean decomposition (LMD), and selected features were fed to the SVM classifier. They achieved a classification accuracy of more than 98.1%. Finally, Bhattacharyya [1] decomposed the EEG signal with the tunable-Q wavelet transform (TQWT) into the number of sub-bands on Q-based entropy and used SVM as a classifier. The authors reported that their model showed the accuracy of 100% and 98.6% for two-class and three-class classifications, respectively.

The general process of the above methods depend on decomposing the EEG signal into several levels, and different features are analyzed from each class to get higher classification result. Unfortunately, there are no available standardized group of features that can accurately describe the signal dynamics. Besides that, incoherent and unnecessary features expand the space of the features, which reduces the accuracy and enhances the risk of overfitting the proposed approach. As a result, those seizure detection approaches are computationally complex and heavily features dependent.

Different authors used DL to detect ES from EEG signals [15,16,17,18,26]. These DL algorithms reduce the feature-dependency problem and provide high accuracy by introducing different network architectures. Ullah [15] proposed an automated system to detect ES using an ensemble of 1-D convolutional neural networks. To split the raw EEG signal, the authors used two data augmentation schemes (e.g., a window size 512 with two different strides 64 and 128). Then the splitting EEG signal was fed into 1-D CNN. Their proposed system was evaluated by a 10-fold CV and achieved an accuracy of 99.1% for three-class problems. Zhao [17] proposed a 1-D DNN to detect ES. In addition, they used the data augmentation technique to enhance the number of EEG samples for training their proposed model. As a result, they achieved the accuracy of 97.63~99.52% for two-class, 96.73~98.06% for three-class, and 93.55% for the complicated five-class problems. To detect ES, Turk [18] proposed a CNN model for learning scalogram image features. The scalogram images have obtained by applying CWT to the input EEG signal. Their proposed model achieved accuracy for two-class, three-class, and five-class 98.5~99.5%, 97.0~99.0%, and 93.6%, respectively.

From the above discussion, it can be observed that many researchers have worked on the detection of ES using various methods, i.e., features extraction approaches, ML, and CNN. Those approaches suffer the problem of weak feature propagation, unable to feature reuse, lack of reducing the number of parameters, etc. However, we proposed a DL architecture comprised of the dense convolutional block (DCB), feature attention modules (FAM), residual blocks (RB), and hypercolumn technique (HT) for the first time to detect ES. Therefore, our model showed outstanding performance for detection of ES in terms of accuracy.

## 3. Dataset

**Figure 1:**
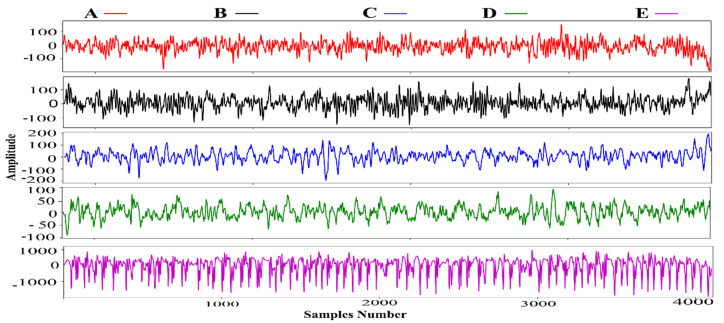
Single sample of each subset (A–E).

In our work, for the classification of ES, we have considered a publicly available EEG dataset provided by the University of Bonn [27], Germany. It is divided into five distinct subsets (sets A–E) or alternately denoted by Z, O, N, F, and S. There are 100 single-channel EEG segments in each subset with a single duration of 23.6 s, where each segment has 4097 samples. In this dataset, both physically fit volunteers and epilepsy patients are used to collecting EEG signals. Sets A and B of EEG signals were collected by placing electrodes on the scalp of volunteers while they were in relaxed conditions with their eyes open and eyes closed. Whereas sets C, D, and E were collected from epilepsy patients. However, sets C and D were recorded during seizure-free intervals. Furthermore, set C is recorded from the hippocampal structure of the opposite hemisphere of the brain, while set D is from the epileptogenic zone. Finally, set E was recorded while seizures appeared. An amplifier having 128 channels was utilized to record the EEG signals setting an average standard reference. The data has been converted from analog to digital form and stored on the disk of a computer. While writing sample rate was set to 173.61 Hz. A bandpass filter was utilized to remove the noise and artifacts setting the low and high cutoff frequencies of 0.53 Hz and 40 Hz, respectively. Table 1 shows the detailed description of the dataset. However, single EEG sample signal from each subset A, B, C, D, and E, is plotted in Figure 1.

## 4. Proposed Methodology

**Figure 2:**
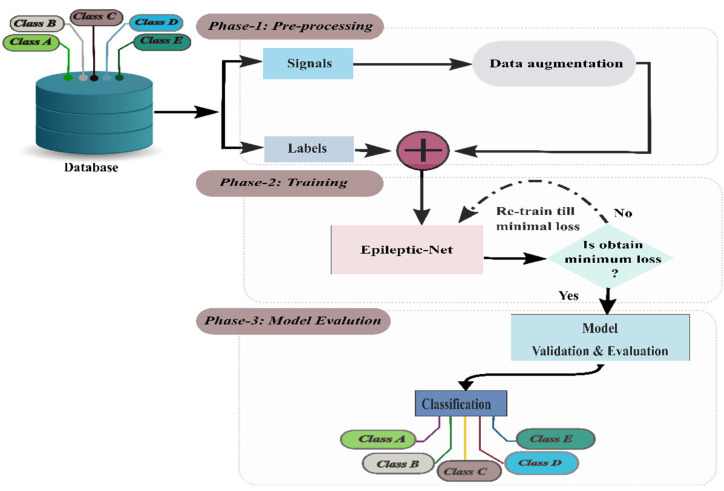
Block diagram of methodological steps to detect the ES.

The block diagram in Figure 2 describes the methodological steps involved with our proposed method to detect the ES. It is divided into three major parts: (i) Input part, which takes an EEG signal, splits it into sub-signals using fixed-size overlapping windows, (ii) training parts, where the preprocessed data has been fed into the proposed model (Epileptic-Net) to train until the minimum loss is obtained, and (iii) evaluate the model to classify/detect ES.

### 4.1. Data Preprocessing

Firstly, the 1-D raw EEG data are normalized, which helps to overcome the model learning problem by making all the data/features values in a similar range. As a result, gradient descents can converge more quickly. However, the total data collected in the University of Bonn EEG dataset is insufficient for training the deep learning model. Therefore, a data augmentation scheme is applied to split the raw EEG signal into several small signals using a fixed size window. Every small signal is treated as an individual instance to learn the Epileptic-Net model. The standard splitting procedure has been used in the existing approaches [15,28,29].

There are 4097 samples in each record in the University of Bonn EEG dataset. Instances are generated from each record by selecting a window size of 1400 and a stride of 150 (10.71% of 1400 with an overlap of 89.29%). Similarly, a total of 2722 instances are produced for each class. Thus, we get 13,610 data for five classes, which are three times more than the original data.

### 4.2. Epileptic-Net

**Figure 3:**
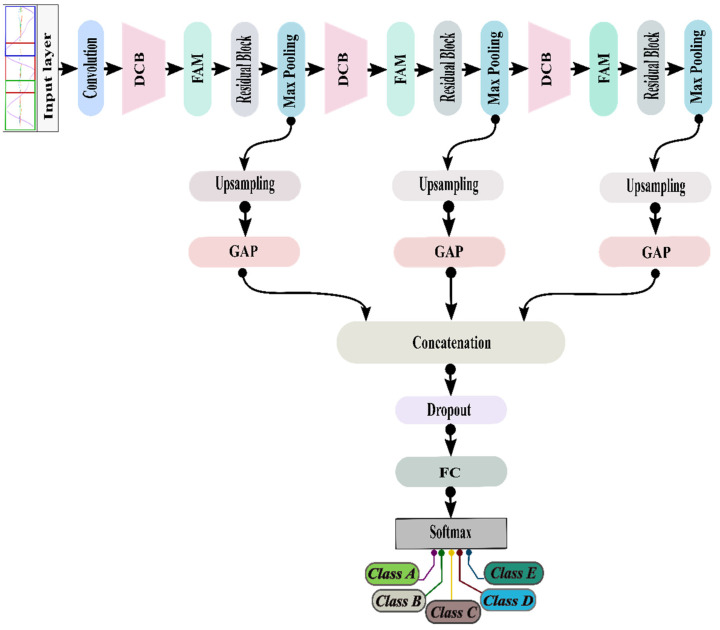
The architecture of our proposed model (Epileptic-Net).

Different studies have shown that deeper convolutional networks are more challenging to train because of the vanishing gradient problem. To avoid this, we used DCB and RB in our model, which helps to alleviate the vanishing gradient, strengthen feature propagation, and encourage feature reuse. However, because of the non-stationary and nonlinear character of the EEG signal, existing methods were unable to provide a sufficient analysis of the signals. Recently, some researchers have also investigated another important issue called the attention mechanism that helps to enhance the performance of CNNs. In our model, we have used FAM that extracts the essential features from the samples. Finally, HT retains the efficient local features extracted from the layers situated at the different levels of the model. So, our proposed Epileptic-Net model synergistically integrates DCB, FAM, RB, and HT to learn powerful feature representations while decreasing the parameters to control the overfitting problem. The architecture of the Epileptic-Net is shown in Figure 3. A brief description of each component of the Epileptic-Net is given below:

#### 4.2.1. Dense Convolutional Block

**Figure 4:**
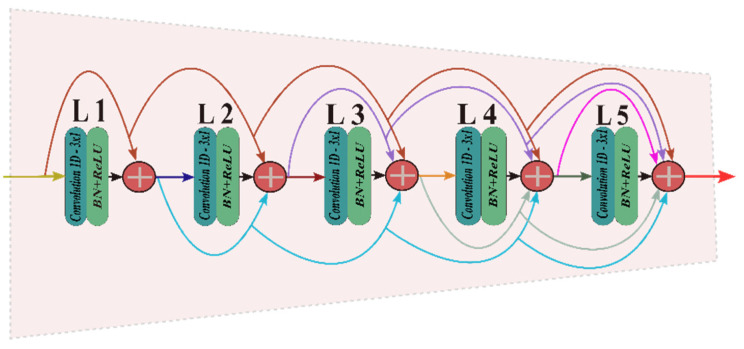
The architecture of DCB.

The dense convolutional block (DCB) consists of convolution layers, batch normalization (BN) layers, and rectified linear units (ReLU) activation functions. After every convolution operation, batch normalization (BN) and ReLU are added. Each layer’s input is a superposition of all the previous layer’s outputs. Each layer’s output is used as the input for all subsequent layers latterly. The features produced by all convolutional layers are connected with dense connections to fuse the multilayer features. These dense connection helps to utilize all feature data of every layer to get specific semantic features. The $X_{i}^{\mathrm{th}}$ layer can be received in Equation (1). A schematic diagram of the DCB is shown in Figure 4.

X_i_ = H_i_ ([X_0_; X_1_; … X_i−1_]) (1)

where (X_0_, X_1_, …, X_i__−1_) are the features generated in layers (0, 1,…, i − 1). H_i_(⋅) means three consecutive operations: convolution, BN, and ReLU.

#### 4.2.2. Feature Attention Module

**Figure 5:**
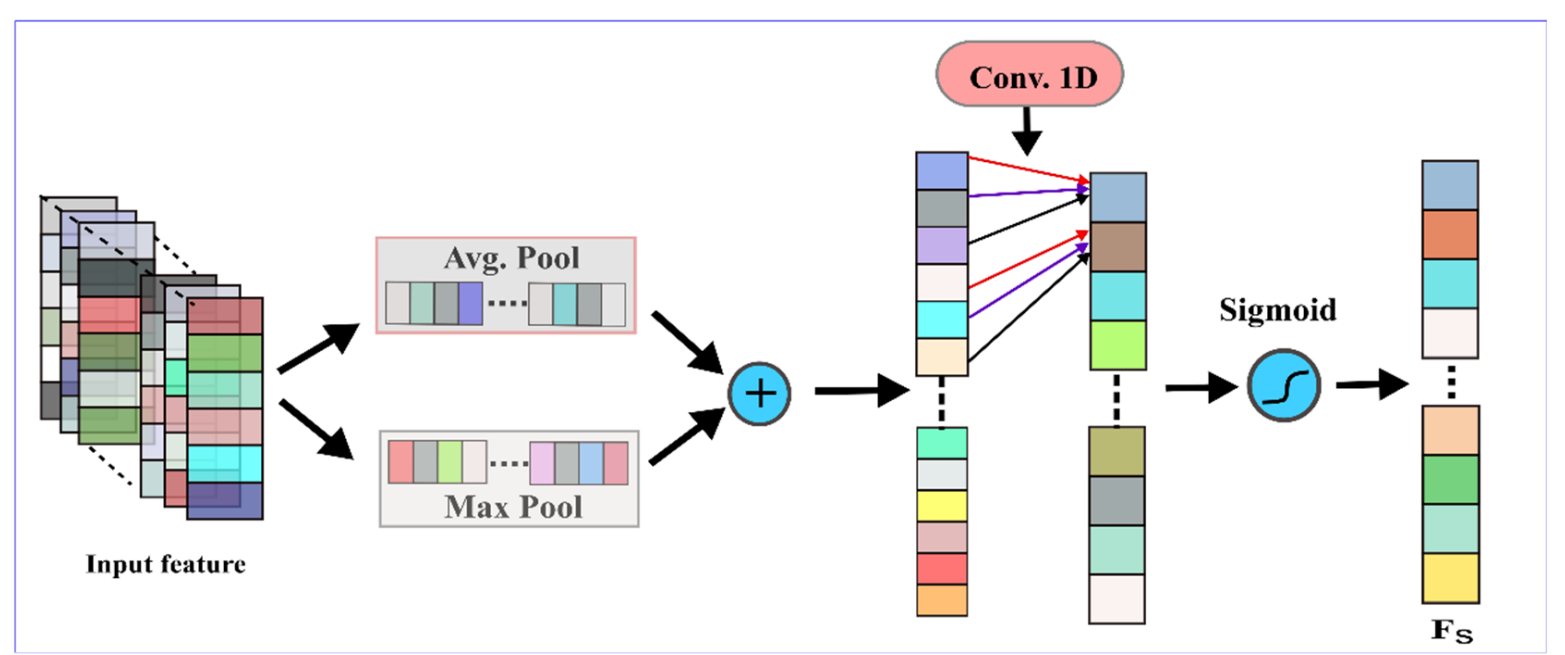
The schematic diagram of the FAM.

The feature attention module (FAM) mainly focuses on the dependence between spatial neighboring values and the noteworthy features on the input data. In other words, the FAM focuses on “where” is an informative part and “which” information is most relevant to an information part. To compute the features attention, we first employ average-pooling and max-pooling operations, then adding them element-wise to yield a set of robust features. Finally, we apply a convolution layer on the concatenated feature descriptor to generate a features attention map F_s_, leading to highlighting or weakening information in the input features. A schematic diagram of the FAM is shown in Figure 5.

Figure 5 shows the kernel feature map is squeezed by utilizing average pooling and max pooling over every 3 × 1 small region. Then, a convolution operation was performed using a single convolution kernel with a filter of size 3 × 1. Lastly, in order to obtain a feature map, a sigmoid activation function is applied to the convolutional operation. The resultant features map is compatible with the input feature map in the spatial dimension. The mathematical expression of the *FAM* part is given as follows:

(2) $$\begin{array}{cc} FAM\left(X\right) & =\sigma (f^{3\times 1}(\left[AvgPool\left(X\right);MaxPool\left(X\right)\right])\\ & =\sigma \left(f^{3\times 1}\left(\left[F_{avg};F_{max}\right]\right)\right) \end{array}$$

*F_avg_* and *F_max_* express average pooling and max pooling operations, and *σ* denotes the sigmoid activation function. Thus, our model can enhance the feature extraction capability by conducting the attention mechanism to spatial dimensions without significantly increasing the number of learning parameters.

#### 4.2.3. Residual Block

**Figure 6:**
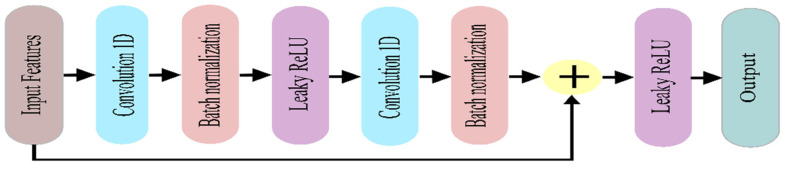
The schematic diagram of residual block.

Simply deep learning networks (LeNet, AlexNet, and VGGNet) usually comprise convolution layers followed by the fully connected layers for classification/regression tasks without any skip/shortcut connection. Each layer sends data to the next layer, and these structures are called sequential networks. With increasing the layer of the sequential network, the difficulty of vanishing or exploding gradient takes place in such networks. To eliminate this problem, residual block in a ResNet allows to skip connections between blocks of convolutional layers, enhances the propagation of gradients, and allows training of even deeper CNNs without having gradient vanishing problems. A residual layer can be expressed as follows:

R(x) = LeakyReLU(x + f(x))(3)

where f(x) is the layer’s output, x is the input, and R(x) is the output of the residual block. In this block, the residual component f(x) is defined as two consecutive copies of a trio of operations: convolution with a filter of size 3 × 1, batch normalization, and Leaky ReLU activation (LeakyReLU activation is not used in second copies). Then, the feature map from f(x) concatenates with input x. Finally, the LeakyReLU activation function is performed on concatenated features. The whole block of R(x) is referred to as a residual block (RB). The schematic diagram of the residual block is shown in Figure 6.

#### 4.2.4. Hypercolumn Technique

For performing classification or recognition, the output of the last layer of a CNN is typically used as the feature representation in the network. However, because of the repeated use of max-pooling and global average pooling, the information in the final FC layer may be too coarse spatially to allow for exact localization. Besides, the earliest layers may be spatially exact, but they contain a lack of semantic information. Therefore, to maintain the balance between spatial precision and the amount of semantic information, the hypercolumn technique (HT) [30] has been adopted. HT is a masking approach that stacks the efficient features gained from the former layers of the CNN model on the original input. HT consists of upsampling, global average pooling (GAP), and concatenation layers. In addition, 50% of dropout is used to prevent overfitting problems.

### 4.3. Hyperparameters

The values of hyperparameters in deep learning are used to control the learning process. The set of hyperparameters used in the proposed model are: (i) the number of epochs, (ii) batch size, (iii) learning rate α, and (v) optimization and loss function. To set the values of these hyperparameters, we initialized the number of epochs = 200 with batch size = 128. If no improvements on the validation loss were found after 30 epochs, we applied an early stopping call back to halt the training process. Initially, we set the learning rate α = 0.001. We, then updated it to 75% of its previous value if the proposed model validation accuracy did not improve after six successive epochs. To minimize the error Adam optimizer [31] has been utilized with setting parameters β1 = 0.9, β2 = 0.999, and ϵ = 1 × 10^−8^. The categorical cross-entropy function is used for determining the error in the optimizer. Recently, it is found out that the cross-entropy technique outperforms other techniques, i.e., classification error and mean squared error [32,33]. The categorical cross-entropy function was used to calculate the error for optimizing the algorithm.

(4) $$X=\frac{1}{b}\sum^{}\left[m\ln x+\left(1-y\right)\ln\left(1-a\right)\right]$$

where *b* is the batch size, *y* is an expected value, and *x* is an actual value from the output layer.

#### 4.3.1. Training

After defining the model hyperparameters, as discussed in previous Section 4.3, the Epileptic-Net was trained on the University of Bonn EEG dataset. Instead of fixed train-test splitting, we used the 10-fold cross-validation (CV) technique to measure the performance of Epileptic-Net. The 10-fold CV strategy divides the whole dataset into ten non overlapped, equal-size folds. It fits the models using an iterative process with nine folds, with the new fold left out for measuring performance (shifting test and train on each iteration). The experiments are done utilizing a Desktop computer with Intel Core i7 3.90 GHz CPU and NVIDIA Titan XP Pro GTX1080Ti 12 GB GPU, 1TB HDD, and 8 GB RAM.

#### 4.3.2. Evaluation Metrics

We have deployed three metrics (Accuracy, F-score, and Cohen’s kappa) to evaluate the recognition model. The accuracy of a recognizer is the percentage of accurate classified cases among the total test cases. Accuracy is a poor metric for real-world classification problems. However, this metric may be acceptable when dealing with classification problems with balanced (or approximately balanced) samples of the classes. In practice, the classes are usually unbalanced. So, we have considered two other evaluation metrics (F-score, Cohen’s kappa) in addition to the accuracy. Suppose TP represents the number of positive cases also classified as positive. In that case, FP represents the number of negative cases classified as positive, TN represents the number of negative cases also classified as negative, and FN represents the number of positive cases classified as negative, then the evaluation metrics are defined as:

(5) $$\mathrm{Accuracy}=\frac{\mathrm{TP}+\mathrm{TN}}{\mathrm{TP}+\mathrm{FP}+\mathrm{TN}+\mathrm{FN}}$$

(6) $$F-\mathrm{score}=2*\frac{\mathrm{Precision}*\;\mathrm{Recall}}{\mathrm{Precision}+\;\mathrm{Recall}}$$

where

(7) $$\mathrm{Recall}=\frac{\mathrm{TP}}{\mathrm{TP}+\mathrm{FN}}$$

(8) $$\mathrm{Precision}=\frac{\mathrm{TP}}{\mathrm{TP}+\mathrm{FP}}$$

We have also considered the reliability metric Cohen’s Kappa (K) to account for how much agreement between the actual and predicted values of a class could be occurred by chance. The value of K ranges from 0 to 1. Thus, the closer K to 1, the more reliable the classifier is.

## 5. Result

This work provides the results for the various experiment cases while performing with the proposed model on the University of Bonn EEG dataset. We performed the four experiment cases: (i) Binary class, (ii) Three class, (iii) Four class, and (iv) Five class. We performed a total of 40 experiments, which have been frequently considered in most of the studies. Among 40 experiments, five experiments have rarely or never been tested. A 10-fold CV procedure was utilized while doing all experiments.

### 5.1. First Experiments: For Binary Class Classification

The first set of experiments was performed with a total of 23 different binary class classifications. We categorized all binary classification into seven different groups. Table 2 shows the performance result of two class ES detection demonstrating superior classification performance and the highest performance of 100% for all groups except the preictal vs. preictal group. Almost all of these binary classification problems have an accuracy of 100%, demonstrating that our proposed model has exceptional classification performance for seizure detection tasks. In addition, our proposed model also yields the best accuracy and Cohen’s kappa (C.K.) for imbalanced class distribution problems (e.g., AB-E, ABC-E, ABCD-E).

### 5.2. Second Experiments: For Three-Class Classification

The potential of the proposed model to distinguish three-class classifications of EEG signals is presented in this section. This case consists of five groups of experiments and Table 3 reports the performance result. The accuracy varies from 99.95% to 100%. In the case of normal vs. preictal vs. ictal (e.g., AB-CD-E, A-D-E) EEG, our model obtains an average accuracy of 100% using the 10-fold cross-validation procedure. The close observations of the performance from Table 3, for normal vs. preictal vs. preictal experiments, normal class (A and B) separately show the same characteristic (accuracy 99.90%) with preictal classes (C, D).

### 5.3. Third Experiments: For Four-Class Classification

The third set of experiments contains three groups of four class classification problems. Table 4 reports the performance of our proposed model for four-class and accuracy varies from 99.91~100%. For example, with the accuracy of normal vs. normal vs. preictal vs. ictal, our model achieved 100%. However, to best our knowledge, only one author [18] has been reported the accuracy (90.50~91.50%) for four-class (e.g., A vs. C vs. D vs. E and B vs. C vs. D vs. E) whereas our model achieved above 99.93% accuracy for those classes. Therefore, it indicates that our proposed model works better for four classes as well.

### 5.4. Fourth Experiments: For Five-Class Classification

Our proposed model achieved a mean accuracy of 99.96% for five-class classifications (e.g., A vs. B vs. C vs. D vs. E). Table 5 shows the fold-wise and average of the 10-fold CV results.

In addition, we have quantified the difference in performance with and without the attention mechanism (FAM) in our model, hereafter called Epileptic-Net-No-FAM. The role of the FAM on the classification performance was assessed by removing the FAM components and repeating the training phase. The removal of FAM with performance consistently lower than 0.8–2.0% with respect to the original model (Epileptic-Net), across all classes (i.e., two, three, four, and five classes). Table 6 shows the number of parameters of the Epileptic-Net model for each class. It has been observed that while the number of classes increases then the number of trainable as well as the total parameters also increases.

Table 7 represents the runtime comparison of the proposed Epileptic-Net model for each class. It shows the training and recognition time in terms of the mean and standard deviation. All the time values were obtained over ten repetitions of the 10-fold CV technique.

**Figure 7:**
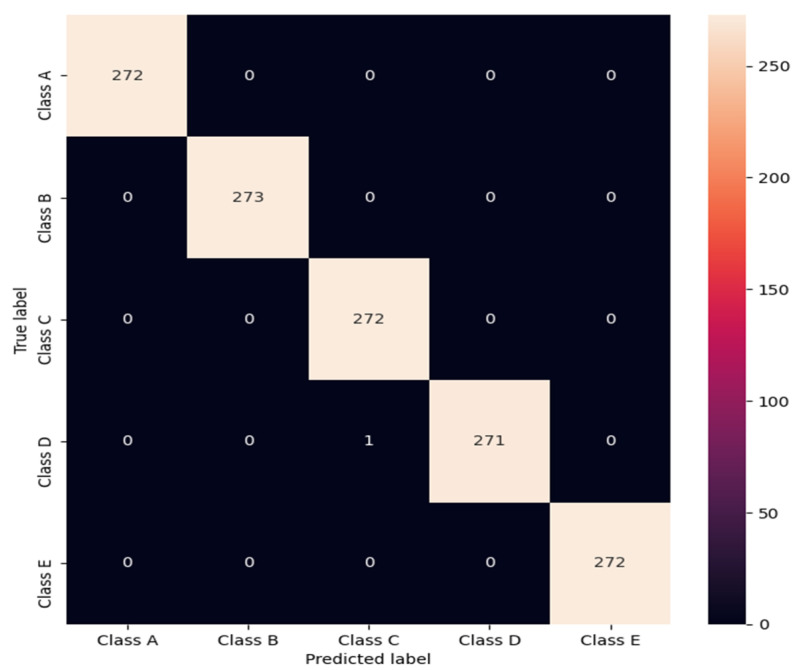
Confusion matrix of the proposed model.

Figure 7 represents a confusion matrix of ES detection, computed over the 10 repetitions format by employing a 10-fold CV procedure. The proposed model predicted the normal and ictal class with 100% accuracy. Thus, the main confusion arises between preictal classes (C and D), with very similar signal patterns.

**Figure 8:**
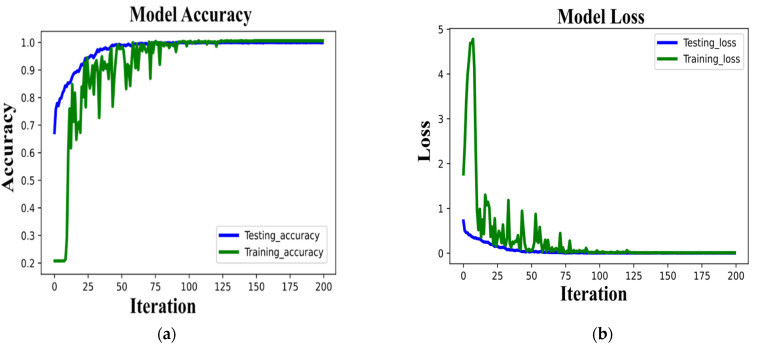
(**a**) The training and testing accuracy of the proposed model. (**b**) The loss history of the proposed model for training and testing set.

Figure 8 shows the accuracy and loss history curve of the Epileptic-Net. It is observed from the graph (Figure 8a) that the training and testing curve of the Epileptic-Net model converges very rapidly within 200 epochs. The training loss is (Figure 8b) comparatively higher than testing loss, which is natural because the training process runs through several phases to learn different patterns of EEG signal.

**Figure 9:**
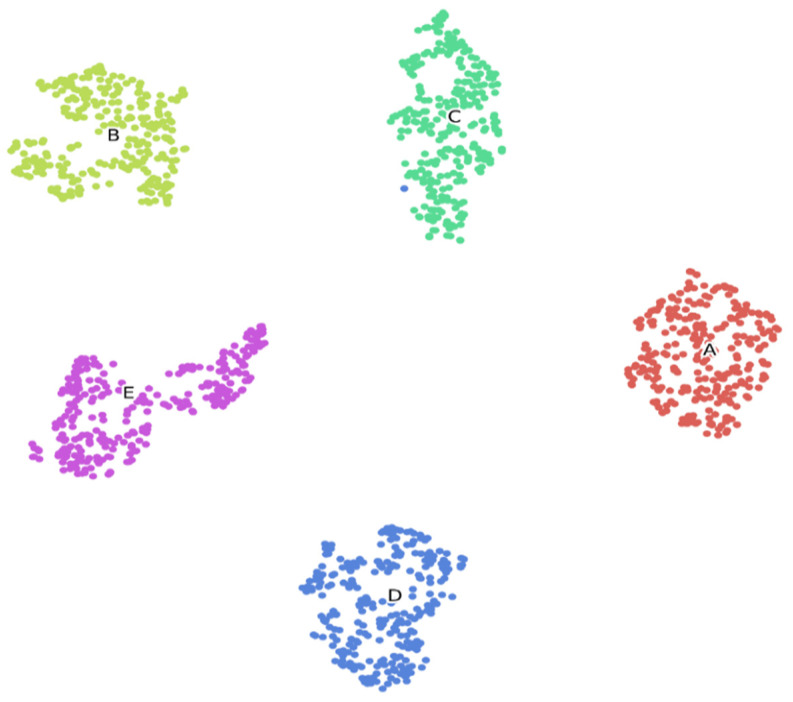
Two-dimensional t-SNE visualization of the learned representations of the Epileptic-Net model for the 10% data of the entire dataset.

How the epileptic-net model represents EEG data in high-dimensional feature space, we visualize these features using the t-SNE algorithm. We have extracted the feature vector from the last FC layer of the Epileptic-Net model and applied t-SNE to map the features onto a 2D space. Figure 9 clearly shows that the EEG data has been separated efficiently, which demonstrates the high generalization capabilities of the proposed Epileptic-Net model.

It is not rational to evaluate the different models using different datasets. Because model performance can vary based on the dataset used for training and testing purposes. To get a robust performance comparison between the proposed models with the existing models, we have utilized the same dataset, the University of Bonn EEG dataset. The performance comparison results are tabulated in Table 8.

## 6. Discussion

This study proposed a deep learning-based CNN model for seizure detection, achieving good performance on the University of Bonn EEG dataset. In the Epileptic-Net model, the discriminative features are extracted using the DCB module with epileptic seizures in EEG signals. In other words, the low-level layers of DCB are utilized to learn the microstructure of EEG signals, which are gone through the subsequent convolutional layers to produce higher-level features. These fine-grained features are adequate for the robust detection by ES. After that, these EEG features are passed into the FAM that only focuses on the informative and relevant part of the EEG signal to detect ES. A total of three RB is introduced, which includes skip-connections between blocks of convolutional layers, enhances the propagation of gradients, and allows the training of even deeper CNNs without gradient vanishing problems. Finally, HT is used to stack the efficient features gained from the previous layers of the proposed model on the EEG signals.

The majority of available epilepsy detection algorithms extracted features by hand-craft engineering, selected the most relevant features by features selection technique, and then fed them into the classifier for ES classification. However, in this work, the feature extraction was performed automatically by the proposed Epileptic-Net model. Furthermore, the proposed model requires the EEG signals as input to produce the corresponding categorization results.

The close observation of the achieved performance evaluation by our proposed Epileptic-Net model is presented from Table 2, Table 3, Table 4 and Table 5 on the University of Bonn EEG dataset for the different types of classes (i.e., two, three, four, and five). In the existing studies, different authors considered various types of binary class classification whereas, we have considered a total of 23 (Table 2) different binary class classifications to validate our Epileptic-Net model. Our Epileptic-Net model has achieved an accuracy of 91.00~100%, F-score of 99.05~100%, and Cohen’s Kappa (C.K.) of 99.00~100%. The author Ullah [15] performed a total of 15 types of binary classification. They proposed a system that was based on an ensemble of pyramidal 1-D CNN models. The authors claimed that the system provides 99.1% to 100% accuracy in all binary cases. The author Ömer [18] performed a total of 10 types of binary classification. They converted raw EEG signals into 2D frequency-time scalograms by using CWT and proposed a CNN model to perform classification. They reported that their proposed CNN model achieved accuracy which varies from 80.00% to 100% for binary class classification. The author Mahfuz [35] used CWT to convert the time-domain signal to a time-frequency image and proposed deep CNN to evaluate their performance where images were used as an input. They also performed 15 types of binary classification and showed that the classification accuracy for binary class varies from 98.13% to 100%.

For the three-class classification problem, we have considered a total of 11 types of classification problems (Table 3). We can see from Table 3, the accuracy, F-score, and Cohen’s Kappa (C.K.) of are 99.90~100%, 99.89~100%, and 99.85~100%, respectively. The author Ömer [18] performed a total of 10 types of three classifications and accuracy varies from 89.71% to 99.66% using CWT and CNN. The authors [1,15,17,36] performed only one type of three-class classification (AB vs. CD vs. E) and achieved less than or equal to 100% accuracy. The author reported 98.60% accuracy using TQWT and SVM [1], using pyramidal 1-D CNN [15] achieved 99.1%, using DNN [17] reported 93.00% and using higher-order statistics, and DNN [36] achieved 100% accuracy. For the four-class classification problem, the author [18] performed two types of four-class (i.e., A vs. C vs. D vs. E, B vs. C vs. D vs. E) classification problems and achieved an accuracy of 90.50% and 91.50%, using CWT and CNN, respectively. Whereas, we have considered a total of five types of four-class classification (Table 4) and our Epileptic-Net has achieved an accuracy of 99.39~100%, F-score of 99.90~100%, and Cohen’s Kappa (C.K.) of 99.90~100%. Lastly, we have performed a five-class classification (Table 5). Our Epileptic-Net model shows the highest accuracy of 99.96%, compared with other works [17,34,36]. The other authors showed a classification performance of 93.66% using DNN [17], 97.20% using higher-order statistics and DNN [34], and 99.88% using optimized variational mode decomposition and reduced deep convolutional neural network [34], respectively.

The performance comparison of the proposed model and those of other existing methods are presented in Table 8 using the same dataset. It has been shown from Table 8 that 35 different two-class, three-class, four-class, and five-class experiments are constructed to evaluate the performance of the proposed model. The proposed method achieved the highest accuracy among the classification problems compared to other models with 10-fold CV. It is seen that the accuracy for C vs. D has changed drastically and improved its classification accuracy by more than 19%. Furthermore, the proposed Epileptic-Net model also showed better performance for the four-class classification and improved its accuracy by 9% more than the existing method. It is more challenging to categorize classification problems with five-class than the classification problems of binary and ternary class. Hence it is necessary to distinguish between EEG signals belonging to the same class (e.g., sets A and B are normal; sets C and D are interictal). The proposed model reported an accuracy of 99.96% for five-class classification problem which is the highest result to date.

## 7. Conclusions

A deep learning-based convolutional neural network architecture is proposed which includes dense convolution block, residual block, and attention mechanism. The proposed model deals with the two-class, three-class, four-class, and five-class classification problems of the publicly available University of Bonn dataset. The raw EEG data has been split into multiple sub-instances using the data augmentation method before being fed to the proposed Epileptic-Net model. The accuracy of the proposed model has significantly improved on various EEG classification problems than the state-of-art works. In conclusion, according to different classification results, the proposed model can diagnose seizures of epilepsy patients with reliable accuracy which raises the confidence of neurologists while diagnose. Although our proposed Epileptic-Net model shows high performance with data augmentation but its performance slightly decreases without augmentation. Furthermore, due to the limited labeled EEG samples and the difficulty of labeling EEG samples, as future work, with the addition of a robust data enhancement algorithm along with the Epileptic-Net can be used to the diagnosing and prediction of other brain diseases such as Parkinson’s, sleep disorders, and eye-blinking. In addition, it is worth mentioning that the proposed model (Epileptic-Net) can be validated on the other datasets also and we will consider a comprehensive study in this issue for future investigations.

## Figures and Tables

**Table 1:** Explanations of the dataset used in this study.

Set	Recording Stage	Electrode Type	Total No. of Segments	Duration of Segments	Predetermined Class
Z or A	Open eyes	Surface	100	23.6 s	normal or healthy
O or B	Closed eyes	Surface	100	23.6 s	normal or healthy
N or C	From hippocampal half sphere	Intracranial	100	23.6 s	preictal or seizure-free
F or D	From epileptic zone	Intracranial	100	23.6 s	preictal or seizure-free
S or E	During seizure	Intracranial	100	23.6 s	ictal or seizure

**Table 2:** Performance result of a binary class with 10-fold CV, here Fi means ith fold and all results are in (%).

Group	Class	F1	F2	F3	F4	F5	F6	F7	F8	F9	F10	Average (%)		
												Acc.	F-Score	C.K.
normal vs. normal	A vs. B	100	100	100	100	100	100	100	100	100	100	100	100	100
normal vs. preictal	A vs. C	100	100	100	100	100	100	100	100	100	100	100	100	100
	A vs. D	100	100	100	100	100	100	100	100	100	100	100	100	100
	B vs. C	100	100	100	100	100	100	100	100	100	100	100	100	100
	B vs. D	100	100	100	100	100	100	100	100	100	100	100	100	100
	AB vs. CD	100	100	100	100	100	100	100	100	100	100	100	100	100
normal vs. ictal	A vs. E	100	100	100	100	100	100	100	100	100	100	100	100	100
	B vs. E	100	100	100	100	100	100	100	100	100	100	100	100	100
	AB vs. E	100	100	100	100	100	100	100	100	100	100	100	100	100
normal vs. preictal and ictal	AB vs. CDE	100	100	100	100	100	100	100	100	100	100	100	100	100
preictal vs. preictal	C vs. D	98	98.20	98.20	98.60	99	99	100	100	100	100	99.10	99	99
preictal vs. ictal	C vs. E	100	100	100	100	100	100	100	100	100	100	100	100	100
	D vs. E	100	100	100	100	100	100	100	100	100	100	100	100	100
	CD vs. E	100	100	100	100	100	100	100	100	100	100	100	100	100
non-ictal vs. ictal	AC vs. E	100	100	100	100	100	100	100	100	100	100	100	100	100
	AD vs. E	100	100	100	100	100	100	100	100	100	100	100	100	100
	BC vs. E	100	100	100	100	100	100	100	100	100	100	100	100	100
	BD vs. E	100	100	100	100	100	100	100	100	100	100	100	100	100
	ABC vs. E	100	100	100	100	100	100	100	100	100	100	100	100	100
	ACD vs. E	100	100	100	100	100	100	100	100	100	100	100	100	100
	ABD vs. E	100	100	100	100	100	100	100	100	100	100	100	100	100
	BCD vs. E	100	100	100	100	100	100	100	100	100	100	100	100	100
	ABCD vs. E	100	100	100	100	100	100	100	100	100	100	100	100	100

**Table 3:** Performance result of three class with 10-fold CV, here Fi means ith fold and all results are in (%).

Group	Class	F1	F2	F3	F4	F5	F6	F7	F8	F9	F10	Average (%)		
												Acc.	F-Score	C.K.
normal vs. preictal vs. ictal	A vs. C vs. E	100	100	100	100	100	100	100	100	100	100	100	100	100
	A vs. D vs. E	100	100	100	100	100	100	100	100	100	100	100	100	100
	B vs. C vs. E	100	100	100	100	100	100	100	100	100	100	100	100	100
	B vs. D vs. E	100	100	100	100	100	100	100	100	100	100	100	100	100
	AB vs. CD vs. E	100	100	100	100	100	100	100	100	100	100	100	100	100
normal vs. normal vs. preictal	A vs. B vs. C	99.50	100	100	100	100	100	100	100	100	100	99.95	99.92	99.90
	A vs. B vs. D	99.80	100	100	100	100	100	100	100	100	100	99.98	99.95	99.97
normal vs. normal vs. ictal	A vs. B vs. E	100	100	100	100	100	100	100	100	100	100	100	100	100
normal vs. preictal vs. preictal	A vs. C vs. D	99.40	100	100	100	100	99.6	100	100	100	100	99.90	99.89	99.85
	B vs. C vs. D	99.40	100	100	100	100	100	100	99.6	100	100	99.90	99.98	99.90
preictal vs. preictal vs. ictal	C vs. D vs. E	100	100	100	100	99.89	100	100	100	100	100	99.98	99.97	99.96

**Table 4:** Performance result of four-class with 10-fold CV, here Fi means ith fold and all results are in (%).

Group	Class	F1	F2	F3	F4	F5	F6	F7	F8	F9	F10	Average (%)		
												Acc.	F-Score	C.K.
normal vs. normal vs. preictal vs. preictal	A vs. B vs. C vs. D	99.86	99.88	99.90	99.90	99.90	100	99.90	99.90	99.90	100	99.91	99.92	99.95
normal vs. normal vs. preictal vs. ictal	A vs. B vs. C vs. E	100	100	100	100	100	100	100	100	100	100	100	100	100
	A vs. B vs. D vs. E	100	100	100	100	100	100	100	100	100	100	100	100	100
normal vs. preictal vs. preictal vs. ictal	A vs. C vs. D vs. E	100	100	99.56	100	100	100	100	100	100	100	99.95	99.93	99.91
	B vs. C vs. D vs. E	100	99.30	100	100	100	100	100	100	100	100	99.93	99.90	99.91

**Table 5:** Performance result of five-class with 10-fold CV, here Fi means ith fold, and all result are in (%).

Class	F1	F2	F3	F4	F5	F6	F7	F8	F9	F10	Average (%)		
											Acc.	F-Score	C.K.
A vs. B vs. C vs. D vs. E	100	100	100	100	99.58	100	100	100	100	100	99.96	99.91	99.85

**Table 6:** Number of parameters of Epileptic-Net model for each class.

Model	Number of Class	Trainable Parameters	Non-Trainable Parameters	Total Parameters
**Epileptic-Net**	Two	318,932	3968	322,900
	Three	318,997	3968	322,965
	Four	319,062	3968	323,030
	Five	319,127	3968	323,095

**Table 7:** Runtime comparison of Epileptic-Net model for each class.

Model	Number of Class	Training Time (s)	Recognition Time (s)
**Epileptic-Net**	Two	506.85 ± 10.56	0.00051 ± 0.000028
	Three	666.48 ± 08.21	0.00077 ± 0.000024
	Four	1010.16 ± 09.80	0.00101 ± 0.000022
	Five	1575.09 ± 12.28	0.001295 ± 0.000028

**Table 8:** Summary of the previous studies and comparative accuracy of the proposed method with the existing methods on the same dataset.

T.C.	T.E.	S.A.	Methodology	E.A. (%)	P.M.A. (%)
Two	A vs. B	Ömer et al. (2019) [18]	CWT and CNN	95.50	**100**
	A vs. C	Ömer et al. (2019) [18]	CWT and CNN	96.50	**100**
	A vs. D	Ömer et al. (2019) [18]	CWT and CNN	100	**100**
	A vs. E	Sahani et al. (2021) [34]	OVMD and DCNN	100	**100**
	B vs. C	Ömer et al. (2019) [18]	CWT and CNN	99.00	**100**
	B vs. D	Ömer et al. (2019) [20]	CWT and CNN	100	**100**
	B vs. E	Sahani et al. (2021) [34]	OVMD and DCNN	100	**100**
	C vs. D	Ömer et al. (2019) [18]	CWT and CNN	80.00	**99.1**
	C vs. E	Sahani et al. (2021) [34]	OVMD and DCNN	100	**100**
	D vs. E	Sahani et al. (2021) [34]	OVMD and DCNN	100	**100**
	AB vs. E	Sahani et al. (2021) [34]	OVMD and DCNN	100	**100**
	AC vs. E	Ullah et al. (2018) [15]	CNN and M-V	99.70	**100**
	AD vs. E	Mahfuz et al. (2021) [35]	FT-VGG16+CWT	98.13	**100**
	BC vs. E	Mahfuz et al. (2021) [35]	FT-VGG16+CWT	99.30	**100**
	CD vs. E	Sahani et al. (2021) [34]	OVMD and DCNN	100	**100**
	AB vs. CD	Ullah et al. (2018) [15]	CNN and M-V	99.98	**100**
	AB vs. CDE	Ullah et al. (2018) [15]	CNN and M-V	99.95	**100**
	ABC vs. E	Ullah et al. (2018) [15]	CNN and M-V	99.97	**100**
	ACD vs. E	Ullah et al. (2018) [15]	CNN and M-V	99.80	**100**
	BCD vs. E	Ullah et al. (2018) [15]	CNN and M-V	99.30	**100**
	ABCD vs. E	Mahfuz et al. (2021) [35]	FT-VGG16+CWT	100	**100**
Three	A vs. B vs. C	Ömer et al. (2019) [18]	CWT and CNN	95.00	**99.95**
	A vs. B vs. D	Ömer et al. (2019) [18]	CWT and CNN	96.67	**99.98**
	A vs. B vs. E	Ömer et al. (2019) [18]	CWT and CNN	95.67	**100**
	A vs. C vs. D	Ömer et al. (2019) [18]	CWT and CNN	88.00	**99.9**
	A vs. C vs. E	Sahani et al. (2021) [34]	OVMD and DCNN	100	**100**
	B vs. C vs. D	Ömer et al. (2019) [18]	CWT and CNN	91.33	**99.9**
	B vs. C vs. E	Sahani et al. (2021) [34]	OVMD and DCNN	100	**100**
	B vs. D vs. E	Sahani et al. (2021) [34]	OVMD and DCNN	100	**100**
	C vs. D vs. E	Ömer et al. (2019) [18]	CWT and CNN	89.00	**99.9**
	A vs. D vs. E	Sahani et al. (2021) [34]	OVMD and DCNN	100	**100**
	AB vs. CD vs. E	Sahani et al. (2021) [34]	OVMD and DCNN	100	**100**
Four	A vs. C vs. D vs. E	Ömer (2019) et al. [18]	CWT and CNN	90.50	**99.95**
	B vs. C vs. D vs. E	Ömer (2019) et al. [18]	CWT and CNN	91.50	**99.93**
Five	A vs. B vs. C vs. D vs. E	Sahani et al. (2021) [34]	OVMD and DCNN	99.88	**99.96**
		Sharma et al. (2020) [36]	TOC and DNN	97.20	
		Zhao et al. (2019) [17]	DNN	93.66	

**T.C.**: Type of class; **T.E.**: Type of experiments; **S.A.**: State-of-the-art; **E.A.**: Existing accuracy; **P.M.A.**: Proposed method accuracy.

## Data Availability

The University of Bonn EEG database can be found at the following website: http://epileptologie-bonn.de/cms/upload/workgroup/lehnertz/ (accessed on 28 November 2021).
